# In Vitro Effect of *Eucalyptus* Essential Oils and Antiseptics (Chlorhexidine Gluconate and Povidone-Iodine) against Bacterial Isolates from Equine Wounds

**DOI:** 10.3390/vetsci11010012

**Published:** 2023-12-26

**Authors:** José Pimenta, Carla Dias, Mário Cotovio, Maria José Saavedra

**Affiliations:** 1 Department of Veterinary Sciences, Antimicrobials, Biocides & Biofilms Unit (A2BUnit), University of Trás-os-Montes and Alto Douro, 5000-801 Vila Real, Portugal; josepimenta@utad.pt (J.P.); cdias@utad.pt (C.D.); mcotovio@utad.pt (M.C.); 2 CECAV—Veterinary and Animal Research Center and Associate Laboratory for Animal and Veterinary Sciences (AL4AnimalS), University of Trás-os-Montes e Alto Douro, 5000-801 Vila Real, Portugal; 3 CIVG—Vasco da Gama Research Center/EUVG–Vasco da Gama University School, 3020-210 Coimbra, Portugal; 4 CITAB—Centre for the Research and Technology of Agro-Environmental and Biological Sciences and Institute for Innovation, Capacity Building and Sustainability of Agri-Food Production (Inov4Agro), University of Trás-os-Montes and Alto Douro, 5000-801 Vila Real, Portugal; 5 Faculty of Veterinary Medicine, Lusófona University, Campo Grande 376, 1749-024 Lisbon, Portugal

**Keywords:** essential oils, *Eucalyptus*, antiseptics, povidone-iodine, chlorhexidine, equine

## Abstract

**Simple Summary:**

Antiseptics have been the most used drugs for wound management in order to reduce the likelihood of infection. However, recent studies have reported the resistance of some strains against antiseptics, which impacts their efficacy, and drawn attention to the impact that the residues of these drugs have on the environment. The aim of this study was to compare the antimicrobial efficacy of antiseptics and eucalyptus essential oils in bacterial strains from horse’s wounds. Twelve *Escherichia coli*, eight *Staphylococcus aureus*, two *Staphylococcus pseudintermedius*, one *Staphylococcus vitulinus* and one *Staphylococcus saprophyticus* isolates, originating from different equine wounds, were used. Through the Kirby-Baüer method, the effect of *Eucalyptus radiata* essential oil, *Eucalyptus globulus* essential oil and antiseptics (povidone-iodine and chlorhexidine gluconate) against the isolated strains was evaluated. *E. radiata* and the mixture of *E. radiata* and *E. globulus* had a statistically significant better inhibitory effect against the *Escherichia coli* strains than antiseptics. Regarding most of the Gram-positive strains tested, *E. globulus* had a better effect compared to *E. radiata*. Chlorhexidine gluconate had a statistically significant better inhibitory effect than povidone-iodine against both Gram-positive and Gram-negative strains. The antibacterial efficacy of essential oils makes these natural compounds potential substitutes for antibiotics and antiseptics, reducing both resistance and the negative environmental impact.

**Abstract:**

Considering the increasing antibiotics resistance, there has been a propensity to replace them with antiseptics when it comes to wound management and treatment. Nevertheless, in recent years, there have been reports regarding resistance to antiseptics by some bacterial strains. There is also concern about the environmental impact of these substances. The aim of this study was to compare the antimicrobial efficacy of antiseptics and eucalyptus essential oils on bacterial strains from horse’s wounds. We used twelve *Escherichia coli*, eight *Staphylococcus aureus*, two *Staphylococcus pseudintermedius*, one *Staphylococcus vitulinus* and one *Staphylococcus saprophyticus* strains from equine wounds. The effect of *Eucalyptus radiata* essential oil, *Eucalyptus globulus* essential oil, povidone-iodine and chlorhexidine gluconate against the isolated strains was evaluated applying the Kirby-Baüer method. Regarding the *Escherichia coli* strains, *E. radiata* and the mixture of *E. radiata* and *E. globulus* had a better inhibitory effect than antiseptics. *E. globulus* had a better effect against most *Staphylococcus* spp. compared to *E. radiata*. For both Gram-negative and Gram-positive strains tested, chlorhexidine gluconate had a better inhibitory effect than povidone-iodine. The antibacterial efficacy of essential oils highlights their potential to substitute or complement the use of antiseptics and so reduce resistance to antiseptics.

## 1. Introduction

Antimicrobial resistance (AMR) is recognized worldwide as one of the most daunting health problems of all time [1,2]. The ineffectiveness of some of the most commonly used antimicrobials in both human and veterinary medicine poses challenges in infection control that could endanger the lives of people and animals [3,4]. This reality highlights the importance of continuous evaluation regarding therapy efficacy used in infection control through the assessment of the resistance profiles of isolated bacteria. Furthermore, it encourages researchers to search for new effective solutions to circumvent this problem.

Wound management is one of the most common procedures in equine clinical practice [5]. Wounds can be associated with some complications, such as delayed healing and infection [5]. One of the main goals of wound medical management is to prevent infection, creating an appropriate environment for epithelial recovery and tissue repair [6,7]. To achieve this purpose, topical antimicrobials are often used, sometimes in an indiscriminate way and without proper knowledge of their effectiveness, a practice that strongly contributes to AMR development [8,9,10,11]. Fortunately, most equine practitioners are leaving this kind of approach, and mainly for acute wounds, the first steps include wound cleansing and antiseptic application [5,12].

Antiseptics are a great choice for a first approach, since they have a wide spectrum of action, and due to the several mechanisms on the bacteria structure, they are less likely to develop bacterial resistances [13,14,15]. However, no single antiseptic will ever achieve all the desired requirements for wound management, such as a broad antibacterial spectrum, low cytotoxicity, good activity even in the presence of organic material, low residue leakage into the environment and low resistance rates [12].

Povidone-iodine and chlorhexidine gluconate are two of the most used antiseptics in equine clinical practice [6,7]. Both have broad spectrum activity against Gram-positive and Gram-negative bacteria and other microorganisms [16]. Povidone-iodine has no reports of bacterial resistance; however, it is inactivated in the presence of organic material such as wound debris, highlighting the importance of wound cleansing, and shows some cytotoxicity, which compromises epithelialization [6,11]. Povidone-iodine is reported to be more effective than chlorhexidine gluconate, particularly against methicillin-resistant *Staphylococcus aureus* [16,17,18]. Chlorhexidine gluconate, unlike povidone-iodine, is not affected by organic material [5,6]. Chlorhexidine gluconate has a cytostatic activity at low concentrations and bactericidal activity at high concentrations. However, it has some limitations, since there are reports of bacterial resistances, particularly in Enterobacteriaceae (e.g., *Serratia* spp., *Proteus* spp. and *Pseudomonas* spp.) and Actinobacteria [18,19]. Although it has some cytotoxicity, it is less severe than povidone-iodine [18,19].

It is widely recognized that there is an urgent need to search for new therapies to replace or support the action of currently used antibiotics and antiseptics [17]. Nature itself is a reservoir of natural antimicrobial compounds that could be extracted mainly from plants, with one of the best examples being the essential oils that have received great acceptance in the pharmaceutical industry and consumers in the past years [20,21,22,23]. This fact is highlighted by the World Health Organization, which has reported that natural therapy is gaining increasing influence among the world’s population [1]. 

This new search should have in mind not only the need for effective antibacterial products but also the need of sustainable sources of these products [24]. Environmental concerns are being discussed globally, and the pharmaceutical industry is under intense pressure to address the negative environmental effects that have resulted from the development and use of medications [3,21]. This worry is mostly caused by the amount of waste from the usage of medications that has been discovered in nature, including antiseptic residues. Even with good antimicrobial practices by clinicians, like judicious prescriptions, such residues perpetuate the cycle of antimicrobial resistance and have nefarious consequences for humans, animals, plants and the environment [24].

*Eucalyptus* oil is one of the most studied and most promising essential oils used to treat wound infections [22,23]. This essential oil exhibits antibacterial, antifungal, analgesic and anti-inflammatory properties, thus being considered as a potentially good broad-spectrum antiseptic [22,25]. The main antibacterial compound of eucalyptus essential oil is the terpene 1,8-cineole, also known as eucalyptole, and its concentration varies between eucalypt species [25]. Different mechanisms of action have been reported; however, due to differences in the bacteria structures, Gram-negative bacteria seem to be more resistant to essential oils compared to Gram-positive bacteria [22]. *E. globulus* and *E. radiata* are in greatly demand in pharmacies and biological product stores, since they are low-cost products that can easily be used by humans for multiple purposes [25,26,27,28]. 

This study aimed to evaluate and compare the in vitro antibacterial activity of two antiseptics (povidone-iodine and chlorhexidine gluconate) commonly used in equine practice and two eucalyptus essential oils (extracted from *E. globulus* and *E. radiata*, respectively) on Gram-negative (*Escherichia coli*) and Gram-positive (*Staphylococcus* spp.) bacteria isolated from equine wounds. Our hypothesis is that essential oils could have a similar or even better effect than commonly used antiseptics against Gram-negative and Gram-positive bacteria isolated from equine wounds. 

## 2. Materials and Methods

### 2.1. Animal Selection and Clinical Information

Horses from several regions of Portugal with traumatic wounds that had not been previously treated with antibiotics were selected. Information regarding age, gender, breed and wound localization was collected (Tables S1 and S2). All owners gave their informed consent for the use of their animal’s data.

### 2.2. Bacteria Sample Collection

Microbiological samples were obtained during clinical practice from wounds of different horses. Each wound was washed with a sterile saline solution to remove surface debris, and then, a surgical swab (Medline, Oxon, Hampshire) was taken from the surface of the wound and stored at 4 °C in Stuart’s transport medium (Oxoid, Hampshire, UK) and sent to the Medical Microbiology Laboratory–Antimicrobials, Biocides and Biofilms Unit, Department of Veterinary Sciences, University of Trás-os-Montes and Alto Douro. The entire procedure was conducted in accordance with the European Animal Welfare Directive (Directive 98/58/CE).

### 2.3. Sample Processing

Samples were cultured in Brain Heart Infusion broth (BHI; Oxoid) and incubated at 35 ± 2 °C for 24 h. After this period, inocula with turbidity were considered positive for bacterial growth and selective, and differential growth media were used for the isolation process. For Gram-negative bacteria, we used GSP *Pseudomonas aeromonas* Selective Agar (Merck, Taufkirchen, Germany), Chromocult Coliform Agar (Oxoid, Hampshire, UK) and MacConkey (Oxoid, Hampshire, UK); for Gram-positive bacteria, we used Baird Parker Agar (Oxoid, Hampshire, UK) and Manitol Salt Agar (Oxoid, Hampshire, UK). Bacterial colonies with different morphophysiological characteristics were collected from each medium, and successive bacterial transferences into other differential growth media were performed until a pure culture was obtained; after which, the bacteria were identified using the procedure mentioned above.

### 2.4. Bacteria Identification

All the isolated bacteria were identified by the automated Vitek^®^ 2 Compact System (BioMerieux, Paris, France) using the Vitek^®^ 2 ID card for Gram-negative and Gram-positive bacteria (BioMérieux, Inc., Durham, NC, USA).

### 2.5. Antibacterial Testing

Twelve *Escherichia coli*, eight *Staphylococcus aureus*, two *Staphylococcus pseudintermedius*, one *Staphylococcus vitulinus* and one *Staphylococcus saprophyticus* strains were used during this study. 

Povidone-iodine 10% (Aga, Lisbon, Portugal) and chlorhexidine gluconate 4% (Aga, Lisbon, Portugal) were commercially acquired from a local pharmacy (Vila Real, Portugal), as well as essential oils from *E. globulus* (0.36 mg/µL) (Biover, Nazareth, Belgium) and *E. radiata* (0.36 mg/µL) (Biover). According to the manufacturer, both essential oils were obtained by hydrodistillation of the leaves and small branches of the tree being rich in both 1,8 cineol, α-terpineol and citrals.

The tested bacteria were enriched after overnight culture in BHI Agar (Oxoid) at 35 ± 2 °C for 24 h. Antibacterial activity was tested using a modification of the disk diffusion Kirby-Baüer method [29]. The suspensions were prepared in sterile saline solution (Braun, Lisbon, Portugal) by adjusting the turbidity to 0.5 McFarland standards and transferred onto in Petri dishes of 90 mm in diameter, which were prepared with 20 mL of Mueller–Hinton Agar (Oxoid, England, UK). Each blank antimicrobial susceptibility disk (Oxoid, England—CT0998B) was impregnated separately with 10 µL of one of the different antiseptics (povidone-iodine at 10%; chlorhexidine gluconate at 4%) or one of the different essential oils (*E. globulus* (0.36 mg/µL); *E. radiata* (0.36 mg/µL)) or with mixtures and placed individually on each inoculated agar. The mixtures used were *E. globulus* + *E. radiata*; chlorhexidine gluconate + *E. radiata*; chlorhexidine gluconate + *E. globulus*; chlorhexidine gluconate + *E. globulus* + *E. radiata*; povidone-iodine + *E. radiata*; povidone-iodine + *E. globulus*; povidone-iodine + *E. radiata* + *E. globulus*. The plates were incubated at 35 ± 2 °C for 24 h. For mixtures (with two or three components), 100 µL of each compound was pipetted into an Eppendorf tube. The mixture was homogenized using a vortex, and 10 µL were subsequently removed and applied to a blank disk.

Blank disks impregnated with an equivalent volume of sodium chloride (NaCl 0.9%) were included as the negative control, and disks with gentamicin (10 µg/disk) (Oxoid, England, UK)) were used as the positive control, according to the antibiotic susceptibility test conducted previously for these isolates (Tables S3 and S4). The antibiotic susceptibility test methodology was the same followed by [9], where the Vitek^®^ 2 Compact System (BioMerieux, Paris, France) was used, and the antimicrobial resistance results were automatically evaluated by Vitek^®^ 2 software, which follows the Clinical and Laboratory Standards Institute (CLSI) guidelines. After incubation, the diameters of the inhibitory zones around the disks were recorded. The tests were conducted three times independently (in triplicate), and the antibacterial activity was expressed as the mean and standard deviation of the observed inhibition diameters (mm).

### 2.6. Statistical Analysis 

Statistical analysis was performed using Jamovi 2.3 version [30] (computer software: https://www.jamovi.org/) (accessed on 1 January 2023). The Shapiro–Wilk test was performed to assess whether the continuous variables followed the normal distribution. To evaluate and compare the antibacterial effect of the different antiseptics, the ANOVA and Tukey’s post hoc tests were performed. The level of significance was defined as *p* < 0.05. 

## 3. Results

### 3.1. Clinical Information

The study group included 13 horses with an average age of 11.2 ± 5.26 years, the youngest being 4 years old and the oldest 20 years old. Regarding gender, five were female, and eight were male. The group included six pure breed Lusitano horses, four crossbred, two Arabians and one Holsteiner. The wound localization was diverse, with the majority being located on the limbs, namely on the fetlock (three), stifle (one), hock (two), pastern (three) and cannon (three) and only one wound on the croup.

### 3.2. Antimicrobial Activity

Regarding the Gram-negative bacteria, we observed that *E. radiata* and the combination of both essential oils (*E. radiata* + *E. globulus*) had the better antibacterial effect, with statistically significant differences (*p* = 0.001), when compared to the most common antiseptics (chlorhexidine gluconate and povidone-iodine) and with all the other combinations made (Table 1). *E. radiata* also had a significantly better effect (*p* = 0.002) than *E. globulus* (Table 1).

Chlorhexidine gluconate had a significantly better effect than povidone-iodine (*p* = 0.02), and although not statistically significant this time (*p* = 0.08), the mean inhibitory diameter of chlorhexidine gluconate was greater when combined with one of the essential oils (15.1 mm compared to chlorhexidine gluconate + *E. radiata* resulting in 17.3 mm or chlorhexidine gluconate + *E. globulus* resulting in 15.4 mm). The combination of chlorhexidine gluconate with each of the essential oils alone had significantly better results that the combination of povidone-iodine with each of the essential oils (*p* = 0.02 for both combinations, CG + ER and CG + EG).

Povidone-iodine had the worst results regarding an antibacterial effect against Gram-negative bacteria. Although not exhibiting statistically significant differences (*p* = 0.62), the mean inhibitory diameter of povidone-iodine improved when mixed with one or both of the essential oils.

The results of the antibacterial activity of povidone-iodine, chlorhexidine gluconate, essential oils and mixtures against each strain of *Escherichia coli* tested (Gram-negative bacteria) are presented in Table 2. Although some differences can be observed between the isolates, *E. radiata* and the combination of *E. globulus* and *E. radiata* had the better antimicrobial effect on most of the isolates. It is also possible to see that, in a few strains, the antiseptics had a better effect than the essential oils. Chlorhexidine gluconate demonstrated greater efficacy than povidone-iodine against all the strains.

Regarding the Gram-positive bacteria, we observed that results were different when compared to the results regarding the Gram-negative bacteria (Table 3). Chlorhexidine gluconate still had a significantly better effect (*p* = 0.005) than povidone-iodine alone; however, this difference was not statistically significant when povidone-iodine was mixed with both essential oils, which improved its antibacterial effect (*p* = 0.09) (Table 3). No significant differences were noted on the antibacterial effect between commonly used antiseptics and essential oils or mixtures of them, with the exception of *E. radiata*, which had a significant lower effect than chlorhexidine gluconate (*p* = 0.008). This time, *E. globulus* had a better effect than *E. radiata*, contrary to the results for the Gram-negative bacteria (*p* = 0.06).

The results of the antibacterial activity of povidone-iodine, chlorhexidine gluconate, essential oils and mixtures against each strain of *Staphylococcus* spp. tested (Gram-positive bacteria) are presented in Table 4. *E. globulus* had the better effect against most of the Gram-positive strains tested when compared to *E. radiata*. In some strains, the combination of both essential oils had a better effect than antiseptics. Chlorhexidine gluconate demonstrated a greater efficacy than povidone-iodine against most strains, with the exception of the isolate *S. aureus E13*.

## 4. Discussion

Equine wounds have a high likelihood of becoming infected, even when all preventive management practices are applied [5]. Due to an erroneous prescription, many Gram-positive and Gram-negative bacteria are being reported as resistant to topical antibiotics, such as *Staphylococcus* spp. and *E. coli*, respectively [18,31]. Wound lavage is the main step for treating and preventing wound infection, with chlorhexidine gluconate and povidone-iodine being the most common antiseptics used for this purpose [32]. However, some reports of bacterial resistance against these antiseptics have emerged, with a special focus on chlorhexidine gluconate [33]. With all these facts in mind, it becomes clear that the search for new effective alternative therapies is urgent.

*E. globulus* and *E. radiata* essential oils have demonstrated good antibacterial effects in several studies similar to the results observed in the present work with strains from equine wounds [22,23,25,26,27,28,34,35,36]. Nicodim et al. observed that *E. globulus* essential oil resulted in a mean diameter of inhibition greater than some commonly used antibiotics against *S. aureus* [20]. Compared to our results, the mean inhibition diameter reported by these authors (20.45 mm) was smaller than what we found in five strains of *S. aureus* tested with *E. globulus* and higher in three other *S. aureus* strains. It was also larger than the inhibitory diameter resulting from povidone-iodine in our *S. aureus* strains. 

The antibacterial potential of eucalyptus essential oils has triggered enormous interest in these compounds. However, although there are many in vitro studies on their antibacterial efficacy, studies on their chemical composition and mechanism of action are scarce [25]. Some studies report that the antibacterial efficacy of eucalyptus essential oils against a wide range of microorganisms stems from their hydrophobicity, which gives them the ability to penetrate the lipid component of the microorganism’s cell membrane, increasing its permeability and thus leading to the loss of cellular components [25,37]. Regarding chemical composition, the main component of eucalyptus essential oil being reported is 1,8-cineole, also known as eucalyptol [25,38]. The concentration of this compound varies according to the species of eucalyptus; however, it has been reported to possess significant antimicrobial activity, although its mechanism of action is not yet fully understood [25,38]. 

The lack of standardized susceptibility testing values for monitoring the development of resistance to antiseptics does not allow us to affirm if the strains tested exhibited resistance or not. However, it was possible to recognize that the antibacterial efficacy of the antiseptics was weaker than the mixture of the two essential oils for the Gram-negative and for some Gram-positive bacteria. Furthermore, regarding the Gram-negative bacteria, the effect of *E. radiata* alone was better than the effect of each antiseptic. These results are in accordance with Karbach et al. [39]. 

Although some studies have proposed a new concept to face bacterial resistance, which includes a combination of conventional antibiotic/antiseptic drugs with essential oils [20,23,35,36], in our study, the interpretation of the results regarding mixtures is not so straightforward. In fact, not all the mixtures seem to bring substantial benefits. For *E. coli.*, the one that stands out is the mixture of both essential oils. For *Staphylococcus* spp., there are two mixtures with overall good results, such as the mixture of chlorhexidine gluconate with *E. globulus* and the mixture of chlorhexidine gluconate with both essential oils.

Similar to our study, Williams et al. investigated the antibacterial efficacy of both antiseptics (chlorhexidine gluconate and povidone-iodine) and of one natural antimicrobial, *Camellia sinensis* (green tea), against commensal bacteria from different animal skins, including *S. aureus* [40]. Some results of the aforementioned study are in accordance with our work, with chlorhexidine gluconate demonstrating a higher efficacy than povidone-iodine, with statistically significant differences. The aforementioned study reported a mean inhibition diameter for chlorhexidine gluconate against *Staphylococcus aureus* strains (24 ± 2.05 mm) that was within the values that we obtained [40]. However, the natural component tested (green tea) was less effective than those antiseptics regarding antimicrobial activity, mainly when compared to chlorhexidine gluconate. Furthermore, no statistically significant differences were observed between green tea and povidone-iodine regarding antimicrobial activity. Both results are not in accordance with our study, since *E. globulus* had a greater effect against *Staphylococcus aureus* than the two antiseptics tested. This lack of consensus between studies highlights the need for more studies on the in vitro and in vivo efficacy of natural components with antimicrobial potential in order to assess which are truly effective [40]. 

Rafferty et al. reported that chlorhexidine gluconate was effective against all *E. coli* strains from canine wounds, disagreeing with the recent reports about resistances to chlorhexidine gluconate [41]. It is also important to highlight that, in our study, for both the Gram-negative and Gram-positive bacteria tested, chlorhexidine gluconate showed better results than povidone-iodine, again contrary to the most recent literature that addresses, with some concern, the observed resistances against chlorhexidine gluconate [41].

Thongrueang et al. studied the in vitro effect of both chlorhexidine gluconate and povidone-iodine against pathogenic bacteria from cows with uterine infection, including *E. coli* strains [42]. Both antiseptics were efficient, and there were no statistically significant differences between them, which is in contrast with our work. However, the authors of the aforementioned study highlighted the cytotoxicity of both antiseptics, which can compromise the recovery of epithelial cells. According to the same authors, chlorhexidine gluconate seems to have less cytotoxicity than povidone-iodine in in vitro studies [42].

In contrast to the findings of the previously mentioned articles, McLure et al. conducted a study on the in vitro effects of chlorhexidine gluconate and povidone-iodine against 33 clinical isolates of *Staphylococcus aureus,* reporting that povidone-iodine exhibited higher antimicrobial activity than chlorhexidine gluconate, a result which contradicts our own research [43]. The authors of the aforementioned study [43] discussed that variations in antibacterial activity between povidone-iodine and chlorhexidine gluconate could be attributed to various factors within the methodology, such as differences in exposure time, dilutions or even variations in the susceptibility of individual strains. However, given the age of their study, we speculate that the disparities compared to our research may be linked to changes in the susceptibility profiles of bacteria. Antimicrobial and biocide susceptibility profiles have likely evolved through genetic mutations, which may explain the differences in the antiseptic resistance pattern presented by our work, where povidone iodine presented more resistances than chlorhexidine gluconate. Hence, this discrepancy between older and more recent studies underscores the importance of ongoing surveillance to assess the effectiveness of antiseptics.

Antibiotics and their residues found in nature are some of the main anthropogenic pollutants of our time [24]. These residues lead to the increased selection of microorganism resistance and the creation of cross-resistance and co-resistance strains [24,44]. This reality is not limited to antibiotics, with antiseptics also being implicated. There have been reports of antiseptics like chlorhexidine gluconate that were detected in water and soils close to hospital settings [45]. The contact of these antiseptics with nature creates exactly the same harmful consequences as antibiotics [45]. 

A possible limitation of this study is related to the composition of the essential oils, since they contain nonpolar compounds [25]. Some of these compounds do not dissolve in liquid solutions, and therefore, their action in an aqueous agar medium (as is the case with the medium used) may be compromised. The authors therefore suggest that their antibacterial activity may be higher than that reported in this study.

More studies about the in vitro and in vivo effects of essential oils, as well as assays regarding minimum inhibitory concentrations, are needed to further characterize their real antibacterial activity and standardize administration methods and effective concentrations. For future studies, experimental studies regarding evaluations of the bacteriostatic or bactericidal activities of essential oils and their combinations with antimicrobial drugs should be performed. The potential synergistic effect that essential oils may have, potentiating the action of antibiotics, could be a way of preventing therapy failure due to antibiotic resistance. 

## 5. Conclusions

A new era for antimicrobial therapy is coming, and urgent search for new therapies is needed. A greater acceptance by consumers and the ready availability of natural sources of compounds with antibacterial properties should be the areas of focus. To the authors’ knowledge, this is the first study that compares the two most common topical antiseptics and two essential oils in strains from equine wounds.

The present results seem to corroborate the initial hypothesis created by the authors, with essential oils possessing similar, and sometimes better, antibacterial effect than antiseptics. *E. globulus* and *E. radiata* essential oils seem to warrant further investigation regarding their antibacterial effects, given the large inhibition diameters presented in the majority of the strains in our work. Particularly, *E. globulus* had a better effect against Gram-positive bacteria (different *Staphylococcus* spp. tested in this study), and *E. radiata* had a better effect against Gram-negative bacteria (*E. coli*). However, contrary to some of the literature, the antiseptics tested in this work seemed to show good antibacterial action. In particular, chlorhexidine gluconate had a better inhibitory effect than povidone-iodine for both Gram-negative and Gram-positive bacteria. Furthermore, since these results highlight the good antibacterial action of essential oils, either in isolation or mixed with antiseptics, future studies on cell lines that evaluate the cytotoxicity and minimum inhibitory concentrations of these agents would be interesting for designing future commercial products to apply to wounds.

## Figures and Tables

**Table 1:** Comparison of the antibacterial activity of various antiseptics and essential oils against the Gram-negative bacteria (*E. coli*).

Antiseptic/Essential Oils	Mean ± Standard Deviation (mm)	Statistical Significance *
ER	27 ± 5.1	**A**
EG + ER	23 ± 5.1	**A**
CG + ER	17.3 ± 5.3	**B**
CG + EG	15.4 ± 2.2	**BC**
CG	15.1 ± 2.1	**BCD**
CG + EG + ER	13.7 ± 4.2	**BCDE**
EG	13.4 ± 2.8	**BCDE**
PI + ER	11.7 ± 3.1	**CDE**
PI + EG + ER	11 ± 2.6	**CDE**
PI + EG	10.7 ± 1	**DE**
PI	10.2 ± 1.2	**E**

ER: *E. radiata*; EG: *E. globulus*; CG: chlorhexidine gluconate; PI: povidone-iodine. * Levels that are not connected by the same letter mean that there is a statistically significant difference between them.

**Table 2:** Comparison of the antibacterial activity of various antiseptics and essential oils against the Gram-negative bacteria (*Escherichia coli*).

Bacterial Stains	Povidone-Iodine (PI)	Chlorhexidine Gluconate (CG)	*Eucalyptus globulus* (EG)	*Eucalyptus* *radiata* (ER)	EG + ER	PI + EG	CG + EG	PI + ER	CG + ER	PI + EG + ER	CG + EG + ER
*E. coli E01*	11.7 ± 0.5	14.7 ± 0.5	11.3 ± 0.5	34.7 ± 0.5	17.7 ± 0.5	10.7 ± 0.5	16.7 ± 0.5	10.3 ± 0.9	14.7 ± 0.5	11.0 ± 0.8	16.3 ± 0.5
*E. coli E02*	11.0 ± 0.8	15.7 ± 0.5	10.7 ± 0.5	21.0 ± 0.8	25.7 ± 0.5	9.7 ± 0.5	15.7 ± 0.5	12.3 ± 0.5	14.7 ± 0.5	11.7 ± 0.5	8.7 ± 0.5
*E. coli E03*	11.7 ± 0.5	16.7 ± 0.5	17.3 ± 0.5	22.3 ± 0.5	18.7 ± 0.5	11.7 ± 0.9	17.7 ± 0.5	15.7 ± 0.5	13.7 ± 0.5	16.3 ± 0.5	14.7 ± 0.5
*E. coli E04*	10.7 ± 0.5	17.3 ± 0.5	10.7 ± 0.5	28.7 ± 0.5	20.7 ± 0.5	10.3 ± 0.9	14.3 ± 0.5	16.7 ± 0.5	12.3 ± 0.5	10.3 ± 0.5	9.7 ± 0.9
*E. coli E05*	9.7 ± 0.5	18.7 ± 0.5	18.7 ± 0.5	20.3 ± 0.5	26.3 ± 0.5	10.7 ± 0.5	14.7 ± 0.5	7.7 ± 0.5	25.7 ± 0.5	7.3 ± 0.5	21.7 ± 0.5
*E. coli E06*	9.7 ± 0.5	14.7 ± 0.5	11.3 ± 0.5	35.3 ± 0.5	20.7 ± 0.5	11.7 ± 0.5	18.7 ± 0.5	9.3 ± 0.9	15.7 ± 0.5	9.0 ± 0.8	14.3 ± 0.5
*E. coli E07*	9.0 ± 0.8	11.7 ± 0.5	11.7 ± 0.5	27.0 ± 0.8	35.7 ± 0.5	10.0 ± 0.5	18.3 ± 0.5	9.7 ± 0.5	16.7 ± 0.5	9.7 ± 0.5	8.7 ± 0.5
*E. coli E08*	10.7 ± 0.5	13.7 ± 0.5	11.3 ± 0.5	30.7 ± 0.5	20.7 ± 0.5	9.7 ± 0.5	15.7 ± 0.5	9.3 ± 0.9	15.7 ± 0.5	11.0 ± 0.8	13.3 ± 0.5
*E. coli E09*	11.0 ± 0.8	11.7 ± 0.5	12.7 ± 0.5	26.0 ± 0.8	18.7 ± 0.5	11.7 ± 0.5	13.7 ± 0.5	11.3 ± 0.5	15.7 ± 0.5	10.7 ± 0.5	9.7 ± 0.5
*E. coli E10*	8.7 ± 0.5	13.7 ± 0.5	16.3 ± 0.5	25.3 ± 0.5	20.7 ± 0.5	9.7 ± 0.9	15.7 ± 0.5	15.7 ± 0.5	16.7 ± 0.5	15.3 ± 0.5	15.7 ± 0.5
*E. coli E11*	8.7 ± 0.5	15.3 ± 0.5	12.7 ± 0.5	30.7 ± 0.5	22.7 ± 0.5	10.3 ± 0.9	11.3 ± 0.5	14.7 ± 0.5	15.3 ± 0.5	11.3 ± 0.5	11.7 ± 0.9
*E. coli E12*	9.7 ± 0.5	16.7 ± 0.5	15.7 ± 0.5	22.3 ± 0.5	27.3 ± 0.5	11.7 ± 0.5	12.7 ± 0.5	8.7 ± 0.5	30.7 ± 0.5	8.0 ± 0.5	19.7 ± 0.5

**Table 3:** Comparison of the antibacterial activity of various antiseptics and essential oils against the Gram-positive bacteria (*Staphylococcus* spp.).

Antiseptic/Essential Oils	Mean ± Standard Deviation (mm)	Statistical Significance *
CG	22.9 ± 6.5	**A**
EG	21.2 ± 8.0	**AB**
CG + EG	17.1 ± 4.5	**ABC**
CG + EG + ER	16.3 ± 6.0	**ABC**
EG + ER	16.1 ± 6.6	**ABC**
PI + EG + ER	14.2 ± 3.3	**ABC**
PI	14.0 ± 4.4	**BC**
PI + EG	13.7 ± 3.6	**BC**
ER	13.6 ± 5.9	**BC**
CG + ER	13.1 ± 4.4	**BC**
PI + ER	10.7 ± 1.1	**C**

ER: *E. radiata*; EG: *E. globulus*; CG: chlorhexidine gluconate; PI: povidone-iodine. * Levels that are not connected by the same letter mean that there is a statistically significant difference between them.

**Table 4:** Mean and standard deviation (millimeters) of the antibacterial activity of essential oils, povidone-iodine and chlorhexidine gluconate and mixtures against the Gram-positive strains.

Bacterial Stains	Povidone-Iodine (PI)	Chlorhexidine Gluconate (CG)	*Eucalyptus globulus* (EG)	*Eucalyptus* *radiata* (ER)	EG + ER	PI + EG	CG + EG	PI + ER	CG + ER	PI + EG + ER	CG + EG + ER
*S. aureus E13*	20.3 ± 0.5	17.7 ± 0.5	41.7 ± 0.5	23.7 ± 0.5	31.3 ± 0.9	17.7 ± 0.5	19.3 ± 0.5	10.3 ± 0.9	10 ± 0.0	15.3 ± 0.5	16.3 ± 0.9
*S. aureus E14*	11.3 ± 0.8	23 ± 0.5	11.0 ± 0.5	11.7 ± 0.8	14.3 ± 0.5	11.7 ± 0.5	19.7 ± 0.5	13.3 ± 0.5	20.7 ± 0.9	21.7 ± 0.5	20 ± 0.0
*S. aureus E15*	19.7 ± 0.5	20.3 ± 0.5	40.7 ± 0.5	15.3 ± 0.5	24.7 ± 0.5	15.7 ± 0.5	19.3 ± 0.5	10.3 ± 0.5	8 ± 0.5	15.7 ± 0.5	30.7 ± 0.5
*S. aureus E16*	11 ± 0.5	17 ± 0.5	35.7 ± 0.5	20.7 ± 0.8	20.3 ± 0.5	20.3 ± 0.5	9 ± 0.5	9 ± 0.5	9.3 ± 0.5	11 ± 0.5	15 ± 0.5
*S. aureus E17*	12.7 ± 0.8	25 ± 0.5	30.0 ± 0.5	22 ± 0.5	16 ± 0.5	12.3 ± 0.5	12.7 ± 0.9	11 ± 0.5	9.0 ± 0.5	10 ± 0.5	18 ± 0.5
*S. aureus E18*	19.7 ± 0.5	20 ± 0.5	42.7 ± 0.5	17.3 ± 0.8	19 ± 0.5	18 ± 0.5	11 ± 0.5	10 ± 0.5	9.0 ± 0.5	10 ± 0.5	18.7 ± 0.8
*S. aureus E19*	9.0 ± 0.5	25.3 ± 0.5	8.0 ± 0.5	9 ± 0.5	10.7 ± 0.5	10.3 ± 0.9	20.7 ± 0.5	10 ± 0.5	14.7 ± 0.5	12.7 ± 0.5	17.7 ± 0.5
*S. aureus E20*	19.7 ± 0.5	30.3 ± 0.5	8.0 ± 0.5	10 ± 0.5	11.7 ± 0.5	12.3 ± 0.5	21.3 ± 0.5	11.3 ± 0.9	17.7 ± 0.5	15.7 ± 0.5	9.0 ± 0.5
*S. pseudintermedius E21*	11.0 ± 0.5	15.3 ± 0.8	9.0 ± 0.5	8.3 ± 0.5	10.7 ± 0.5	9.3 ± 0.9	15.7 ± 0.5	11.3 ± 0.5	12.7 ± 0.5	13.7 ± 0.5	14.7 ± 0.5
*S. pseudintermedius E22*	11.7 ± 0.5	35.3 ± 0.5	10.0 ± 0.5	9 ± 0.5	12.7 ± 0.5	15.3 ± 0.5	22.3 ± 0.5	10.3 ± 0.9	12.7 ± 0.5	16.7 ± 0.5	10.0 ± 0.5
*S. vitulinus E23*	10.0 ± 0.5	15.3 ± 0.5	9.0 ± 0.5	8.3 ± 0.5	10.7 ± 0.5	9.3 ± 0.9	13.7 ± 0.5	11.7 ± 0.5	13.7 ± 0.5	12.7 ± 0.5	17.7 ± 0.5
*S. saprophyticus E24*	11.7 ± 0.5	30.3 ± 0.5	8.0 ± 0.5	8 ± 0.5	10.7 ± 0.5	12.3 ± 0.5	20.3 ± 0.5	10.3 ± 0.9	19.7 ± 0.5	14.7 ± 0.5	8.3 ± 0.5

## Data Availability

Data are contained within the article and Supplementary Materials.

## Supplementary Materials

The following supporting information can be downloaded at https://www.mdpi.com/article/10.3390/vetsci11010012/s1: Table S1: Clinical information on the horses and wounds selected for the study. Information regarding the Gram-negative (*Escherichia coli*) strains; Table S2: Clinical information on the horses and wounds selected for the study. Information regarding the Gram-positive strains; Table S3: Antibiotic susceptibility test results for the *Escherichia coli* strains; Table S4: Antibiotic susceptibility test results for the Gram-positive strains.

## References

[1] World Health Organization and Antimicrobial Resistance. https://www.who.int/health-topics/antimicrobial-resistance.

[2] World Organization for Animal Health-OIE and Antimicrobial Resistance. https://www.woah.org/en/what-we-do/global-initiatives/antimicrobial-resistance/.

[3] Ferri M., Ranucci E., Romagnoli P., Giaccone V. (2017). Antimicrobial Resistance: A Global Emerging Threat to Public Health Systems. Crit. Rev. Food Sci. Nutr..

[4] Palma E., Tilocca B., Roncada P. (2020). Antimicrobial Resistance in Veterinary Medicine: An Overview. Int. J. Mol. Sci..

[5] Theoret C., Schumacher J., Theoret C., Schumacher J. (2017). Equine Wound Management. Equine Wound Management.

[6] Hanson R.R. (2018). Medical Therapy in Equine Wound Management. Vet. Clin. N. Am.-Equine Pract..

[7] Frees K.E. (2018). Equine Practice on Wound Management: Wound Cleansing and Hygiene. Vet. Clin. N. Am.-Equine Pract..

[8] Westgate S.J., Percival S.L., Knottenbelt D.C., Clegg P.D., Cochrane C.A. (2011). Microbiology of Equine Wounds and Evidence of Bacterial Biofilms. Vet. Microbiol..

[9] Afonso A.C., Sousa M., Pinto A.R., Cotovio M., Simões M., Saavedra M.J. (2023). Biofilm Production by Critical Antibiotic-Resistant Pathogens from an Equine Wound. Animals.

[10] Isgren C.M., Williams N.J., Fletcher O.D., Timofte D., Newton R.J., Maddox T.W., Clegg P.D., Pinchbeck G.L. (2022). Antimicrobial Resistance in Clinical Bacterial Isolates from Horses in the UK. Equine Vet. J..

[11] Leise B.S. (2018). Topical Wound Medications. Vet. Clin. N. Am.-Equine Pract..

[12] Jørgensen E., Bjarnsholt T., Jacobsen S. (2021). Biofilm and Equine Limb Wounds. Animals.

[13] Maillard J.Y., Kampf G., Cooper R. (2021). Antimicrobial Stewardship of Antiseptics That Are Pertinent to Wounds: The Need for a United Approach. JAC Antimicrob. Resist..

[14] Alves P.J., Barreto R.T., Barrois B.M., Gryson L.G., Meaume S., Monstrey S.J. (2021). Update on the Role of Antiseptics in the Management of Chronic Wounds with Critical Colonisation and/or Biofilm. Int. Wound J..

[15] Roberts C.D., Leaper D.J., Assadian O. (2017). The Role of Topical Antiseptic Agents within Antimicrobial Stewardship Strategies for Prevention and Treatment of Surgical Site and Chronic Open Wound Infection. Adv. Wound Care.

[16] Bigliardi P.L., Alsagoff S.A.L., El-Kafrawi H.Y., Pyon J.K., Wa C.T.C., Villa M.A. (2017). Povidone Iodine in Wound Healing: A Review of Current Concepts and Practices. Int. J. Surg..

[17] Lachapelle J.M., Castel O., Casado A.F., Leroy B., Micali G., Tennstedt D., Lambert J. (2013). Antiseptics in the Era of Bacterial Resistance: A Focus on Povidone Iodine. Clin. Pract..

[18] Williamson D.A., Carter G.P., Howden B.P. (2017). Current and Emerging Topical Antibacterials and Antiseptics: Agents, Action, and Resistance Patterns. Clin. Microbiol. Rev..

[19] Barrigah-Benissan K., Ory J., Sotto A., Salipante F., Lavigne J.P., Loubet P. (2022). Antiseptic Agents for Chronic Wounds: A Systematic Review. Antibiotics.

[20] Nicodim L., Rapuntean G., Rapuntean S., Chirila F., Nadas G. (2009). Antibacterial Effect of Essential Vegetal Extracts on *Staphylococcus aureus* Compared to Antibiotics. Hort. Agrobot. Cluj.

[21] Teixeira I.D., Carvalho E., Leal E.C. (2023). Green Antimicrobials as Therapeutic Agents for Diabetic Foot Ulcers. Antibiotics.

[22] Azzam N.F.A.E.M. (2020). Antibacterial Effect of Eucalyptus Essential Oil. Indian J. Sci. Technol..

[23] Aleksic Sabo V., Knezevic P. (2019). Antimicrobial Activity of *Eucalyptus camaldulensis* Dehn. Plant Extracts and Essential Oils: A Review. Ind. Crops Prod..

[24] Apreja M., Sharma A., Balda S., Kataria K., Capalash N., Sharma P. (2022). Antibiotic Residues in Environment: Antimicrobial Resistance Development, Ecological Risks, and Bioremediation. Environ. Sci. Pollut. Res..

[25] Luís Â., Duarte A., Gominho J., Domingues F., Duarte A.P. (2016). Chemical Composition, Antioxidant, Antibacterial and Anti-Quorum Sensing Activities of *Eucalyptus globulus* and *Eucalyptus radiata* Essential Oils. Ind. Crops Prod..

[26] Bachir R.G., Benali M. (2012). Antibacterial Activity of the Essential Oils from the Leaves of *Eucalyptus globulus* against *Escherichia coli* and *Staphylococcus aureus*. Asian Pac. J. Trop. Biomed..

[27] Müller-Heupt L.K., Vierengel N., Groß J., Opatz T., Deschner J., von Loewenich F.D. (2022). Antimicrobial Activity of *Eucalyptus globulus*, *Azadirachta indica*, *Glycyrrhiza glabra*, *Rheum palmatum* Extracts and Rhein against *Porphyromonas gingivalis*. Antibiotics.

[28] Pereira V., Dias C., Vasconcelos M.C., Rosa E., Saavedra M.J. (2014). Antibacterial Activity and Synergistic Effects between *Eucalyptus globulus* Leaf Residues (Essential Oils and Extracts) and Antibiotics against Several Isolates of Respiratory Tract Infections (*Pseudomonas aeruginosa*). Ind. Crops Prod..

[29] (1966). Antibiotic Susceptibility Testing by a Standardized Single Disk Method. Am. J. Clin. Pathol..

[30] (2023). The Jamovi Project. *Jamovi* (Version 2.3) [Computer Software]. https://www.jamovi.org.

[31] Harkins C.P., McAleer M.A., Bennett D., McHugh M., Fleury O.M., Pettigrew K.A., Oravcová K., Parkhill J., Proby C.M., Dawe R.S. (2018). The Widespread Use of Topical Antimicrobials Enriches for Resistance in *Staphylococcus aureus* Isolated from Patients with Atopic Dermatitis. Br. J. Dermatol..

[32] Debebe N., Gelaye A., Fesseha H. (2020). Open Wound in Equine and Its Management-Review. CPQ Med..

[33] Aftab R., Dodhia V.H., Jeanes C., Wade R.G. (2023). Bacterial Sensitivity to Chlorhexidine and Povidone-Iodine Antiseptics over Time: A Systematic Review and Meta-Analysis of Human-Derived Data. Sci. Rep..

[34] Mumtaz R., Zubair M., Khan M.A., Muzammil S., Siddique M.H. (2022). Extracts of *Eucalyptus alba* Promote Diabetic Wound Healing by Inhibiting α-Glucosidase and Stimulating Cell Proliferation. Evid.-Based Complement. Altern. Med..

[35] Mulyaningsih S., Sporer F., Reichling J., Wink M. (2011). Antibacterial Activity of Essential Oils from *Eucalyptus* and of Selected Components against Multidrug-Resistant Bacterial Pathogens. Pharm. Biol..

[36] Knezevic P., Aleksic V., Simin N., Svircev E., Petrovic A., Mimica-Dukic N. (2016). Antimicrobial Activity of *Eucalyptus camaldulensis* Essential Oils and Their Interactions with Conventional Antimicrobial Agents against Multi-Drug Resistant *Acinetobacter baumannii*. J. Ethnopharmacol..

[37] Horvathova E., Navarova J., Galova E., Sevcovicova A., Chodakova L., Snahnicanova Z., Melusova M., Kozics K., Slamenova D. (2014). Assessment of Antioxidative, Chelating, and DNA-Protective Effects of Selected Essential Oil Components (Eugenol, Carvacrol, Thymol, Borneol, Eucalyptol) of Plants and Intact Rosmarinus Officinalis Oil. J. Agric. Food Chem..

[38] Goldbeck J.C., do Nascimento J.E., Jacob R.G., Fiorentini Â.M., da Silva W.P. (2014). Bioactivity of Essential Oils from *Eucalyptus globulus* and *Eucalyptus urograndis* against Planktonic Cells and Biofilms of *Streptococcus mutans*. Ind. Crops Prod..

[39] Karbach J., Ebenezer S., Warnke P., Behrens E., Al-Nawas B. (2015). Antimicrobial Effect of Australian Antibacterial Essential Oils as Alternative to Common Antiseptic Solutions against Clinically Relevant Oral Pathogens. Clin. Lab..

[40] Williams J., Lane S., Harniman S. (2016). An In Vitro Investigation into the Efficacies of Chlorhexidine Gluconate, Povidone Iodine and Green Tea (*Camellia sinensis*) to Prevent Surgical Site Infection in Animals. Vet. Nurse.

[41] Rafferty R., Robinson V.H., Harris J., Argyle S.A., Nuttall T.J. (2019). A Pilot Study of the In Vitro Antimicrobial Activity and In Vivo Residual Activity of Chlorhexidine and Acetic Acid/Boric Acid Impregnated Cleansing Wipes. BMC Vet. Res..

[42] Thongrueang N., Liu S.S., Hsu H.Y., Lee H.H. (2022). An In Vitro Comparison of Antimicrobial Efficacy and Cytotoxicity between Povidone iodine and Chlorhexidine for Treating Clinical Endometritis in Dairy Cows. PLoS ONE.

[43] McLure A., Gordon J. (1992). In Vitro Evaluation of Povidone-Iodine and Chlorhexidine against Methicillin-Resistant *Staphylococcus aureus*. J. Hosp. Infect..

[44] Wang W., Weng Y., Luo T., Wang Q., Yang G., Jin Y. (2023). Antimicrobial and the Resistances in the Environment: Ecological and Health Risks, Influencing Factors, and Mitigation Strategies. Toxics.

[45] Basiry D., Heravi N., Uluseker C., Kaster K., Kommedal R., Ozkok P. (2022). The Effect of Disinfectants and Antiseptics on Co- and Cross-Selection of Resistance to Antibiotics in Aquatic Environments and Wastewater Treatment Plants. Front. Microbiol..

