# Supplementary material for: In Vitro Effect of Eucalyptus Essential Oils and Antiseptics (Chlorhexidine Gluconate and Povidone-Iodine) against Bacterial Isolates from Equine Wounds
Source: Vet Sci. 2023 Dec 26;11(1):12. doi: 10.3390/vetsci11010012 (PMC10819342; doi:10.3390/vetsci11010012)
Supplement: Supplementary file 1 [file vetsci-11-00012-s001.zip › vetsci-2732122-supplementary.pdf]

Table S1. Clinical information of the horses and wounds selected for the study. Information regarding Gram-negative (*Escherichia coli*) strains

Isolate	Bacterial strains	Gender	Age	Breed	Lesion localization
E1	*Escherichia coli*	Female	13 years old	Pure Bred Lusitano	Trunk
E2	*Escherichia coli*	Female	13 years old	Pure Bred Lusitano	Trunk
E3	*Escherichia coli*	Female	13 years old	Pure Bred Lusitano	Trunk
E4	*Escherichia coli*	Male	10 years old	Pure Bred Lusitano	Head
E5	*Escherichia coli*	Female	6 years old	Pure Bred Lusitano	Stifle
E6	*Escherichia coli*	Female	12 years old	Cross-bred	Distal limb
E7	*Escherichia coli*	Male	4 years old	Arabian	Distal limb
E8	*Escherichia coli*	Female	4 years old	Cross-bred	Distal limb
E9	*Escherichia coli*	Male	13 years old	Pure Bred Lusitano	Distal limb
E10	*Escherichia coli*	Male	13 years old	Pure Bred Lusitano	Distal limb
E11	*Escherichia coli*	Male	9 years old	Pure Bred Lusitano	Distal limb
E12	*Escherichia coli*	Male	9 years old	Pure Bred Lusitano	Distal limb

Table S2. Clinical information of the horses and wounds selected for the study. Information regarding Gram-positive strains

Isolate	Bacterial strain	Gender	Age	Breed	Lesion localization
**E13**	*Staphylococcus aureus*	Female	12 years old	Pure Bred Lusitano	Distal limb
**E14**	*Staphylococcus aureus*	Female	12 years old	Pure Bred Lusitano	Distal limb
**E15**	*Staphylococcus aureus*	Male	13 years old	Warmblood	Distal limb
**E16**	*Staphylococcus aureus*	Female	1 years old	Cross-bred	Distal limb
**E17**	*Staphylococcus aureus*	Female	12 years old	Pure Bred Lusitano	Distal limb
**E18**	*Staphylococcus aureus*	Male	4 years old	Arabian	Distal limb
**E19**	*Staphylococcus aureus*	Female	4 years old	Cross-bred	Distal limb
**E20**	*Staphyloccus aureus*	Male	18 years old	Pure Bred Lusitano	Neck
**E21**	*Staphylococcus pseudintermedius*	Female	15 years old	Cross-bred	Distal limb
**E22**	*Staphylococcus pseudintermedius*	Female	13 years old	Pure Bred Lusitano	Trunk
**E23**	*Staphylococcus vitulinus*	Male	9 years old	Pure Bred Lusitano	Distal limb
**E24**	*Staphylococcus saprophyticus*	Male	9 years old	Pure Bred Lusitano	Distal limb

Table S3. Antibiotic susceptibility test results for *Escherichia coli* strains.

Bacterial Strains	Antibiotics																		
	TE	DO	C	N	CN	AK	ENR	PFX	F	SXT	MEM	ETP	IPM	AMC	AMP	KF	CL	EFT	CVN	CPD
**E1**	S	S	I	S	S	S	S	S	S	S	S	S	S	S	S	R	R	S	S	S
**E2**	S	S	S	S	S	S	S	S	S	S	S	S	S	S	S	R	R	S	S	S
**E3**	S	S	S	S	S	S	S	S	S	S	S	S	S	S	S	R	R	S	S	S
**E4**	R	I	I	S	S	S	S	S	S	S	S	S	S	S	S	R	R	S	S	S
**E5**	S	S	S	S	S	S	S	S	S	S	S	S	S	S	S	R	R	S	S	S
**E6**	S	S	I	S	S	S	S	S	S	S	S	S	S	S	S	R	R	S	S	S
**E7**	S	S	S	S	S	S	S	S	S	S	S	S	S	S	S	R	R	S	S	S
**E8**	S	S	S	S	S	S	S	S	S	S	S	S	S	S	S	I	R	S	S	S
**E9**	S	S	S	S	S	S	S	S	S	S	S	S	S	S	S	R	R	S	S	S
**E10**	S	S	S	S	S	S	S	S	S	S	S	S	S	S	S	I	R	S	S	S
**E11**	S	S	S	S	S	S	S	S	S	S	S	S	S	S	S	R	R	S	S	S
**E12**	S	S	I	S	S	S	S	S	S	S	S	S	S	S	S	R	R	S	S	S

(TE_{30}): tetracycline; (DO_{30}): doxycycline; (C_{30}): chloramphenicol; (N_{30}): neomycin; (CN_{10}): gentamicin; (AK_{30}): amikacin; (ENR_{5}): enrofloxacin; (PFX_{5}): pefloxacin; (F_{100}): nitrofurantoin; (SXT_{25}): trimethoprim-sulfamethoxazole; (MEM_{10}): meropenem; (ETP_{10}): ertapenem; (IMP_{10}): imipenem; (AMC_{30}): amoxicillin/clavulanate; (AMP_{10}): ampicillin; (KF_{30}): cephalothin; (CL_{30}): cephalexin; (EFT_{30}): ceftiofur; (CVN_{30}): cefovecin; (CPD_{10}): cefpodoxime

Table S4: Antibiotic susceptibility test results for Gram-positive strains.

Bacterial strains	Identification	Antibiotics											
		TE	DO	C	K	N	CN	ENR	PFX	E	DA	F	SXT
**E13**	*Staphylococcus aureus*	S	S	S	S	S	S	S	S	S	S	S	S
**E14**	*Staphylococcus aureus*	S	S	S	S	S	S	S	S	S	S	S	S
**E15**	*Staphylococcus aureus*	S	S	S	S	S	S	S	S	R	R	S	S
**E16**	*Staphylococcus aureus*	S	S	S	S	S	S	S	S	S	S	S	S
**E17**	*Staphylococcus aureus*	S	S	S	S	S	S	S	S	R	R	S	S
**E18**	*Staphylococcus aureus*	S	S	S	S	S	S	S	S	R	R	S	S
**E19**	*Staphylococcus aureus*	S	S	S	S	S	S	S	S	R	R	S	S
**E20**	*Staphyloccus aureus*	R	R	S	R	I	S	R	I	R	R	S	R
**E21**	*Staphylococcus pseudintermedius*	S	S	S	S	S	S	S	S	S	S	S	S
**E22**	*Staphylococcus pseudintermedius*	R	R	S	R	I	S	R	I	R	R	S	R
**E23**	*Staphylococcus vitulinus*	S	S	S	S	S	S	S	S	S	S	S	S
**E24**	*Staphylococcus saprophyticus*	S	S	S	S	S	S	S	S	R	S	S	S

(TE_{30}): tetracycline; (DO_{30}): doxycycline; (C_{30}): chloramphenicol; (K_{30}): kanamycin; (N_{30}): neomycin; (CN_{10}): gentamicin; (ENR_5): enrofloxacin; (PFX_5): pefloxacin; (E_{15}): erythromycin; (DA_2): clindamycin; (F_{100}): nitrofurantoin; (SXT_{25}): trimethoprim-sulfamethoxazole
